# Single Nucleotide Polymorphisms in Distant Kinship Inference and Forensic Genetic Genealogy

**DOI:** 10.3390/ijms27167386

**Published:** 2026-08-18

**Authors:** Denisse Stephania Becerra-Loaiza, Nayeli González-Ortiz, Yolanda Puga-Carrillo, Joel Alberto Aguilar-Velázquez, Itzae Adonai Gutiérrez-Hurtado, José Alonso Aguilar-Velázquez

**Affiliations:** 1 Estancia Posdoctoral Avalada por la Secretaría de Ciencias, Humanidades, Tecnología e Innovación (SECIHTI), Guadalajara 44540, Jalisco, Mexico; 2 Departamento de Morfología, Centro Universitario de Ciencias de la Salud, Universidad de Guadalajara, Guadalajara 44340, Jalisco, Mexico; 3 Instituto de Genética Humana “Dr. Enrique Corona Rivera”, Departamento de Biología Molecular y Genómica, Centro Universitario de Ciencias de la Salud, Universidad de Guadalajara, Guadalajara 44340, Jalisco, Mexico; 4 Doctorado en Genética Humana, Departamento de Biología Molecular y Genómica, Centro Universitario de Ciencias de la Salud, Universidad de Guadalajara, Guadalajara 44340, Jalisco, Mexico; 5 Maestría en Genética Forense e Identificación Humana, Departamento de Morfología, Centro Universitario de Ciencias de la Salud, Universidad de Guadalajara, Guadalajara 44340, Jalisco, Mexico; 6 Departamento de Matemáticas, Universidad Autónoma Metropolitana Unidad Iztapalapa, Mexico City 09340, Mexico; 7 Departamento de Biología Molecular y Genómica, Centro Universitario de Ciencias de la Salud, Universidad de Guadalajara, Guadalajara 44340, Jalisco, Mexico

**Keywords:** single-nucleotide polymorphism, forensic genetic genealogy, investigative genetic genealogy, distant kinship, identity by descent, genetic genealogy database matching, massively parallel sequencing

## Abstract

Forensic genetics is moving from locus-based DNA profiling toward genome-wide inference enabled by high-density single-nucleotide polymorphism (SNP) data. While short tandem repeats remain central to routine human identification, SNP-based technologies and massively parallel sequencing have expanded the analysis of distant kinship through detection of identity-by-descent (IBD) segments and shared autosomal DNA. This narrative review synthesizes the biological basis of SNP-based distant kinship inference, the statistical and computational frameworks used to model genomic relatedness, and the operational transition from relatedness detection to forensic genetic genealogy (FGG). It distinguishes genetic genealogy database matching from formal forensic kinship testing, targeted SNP panels, SNP capture, low-coverage sequencing, Bayesian and machine-learning approaches, and independent forensic confirmation. Applications in criminal investigations, unidentified human remains, historical identifications, and broader relationship-inference contexts are discussed. The review also examines limitations related to recombination, stochastic inheritance, marker density, genotype quality, degraded or mixed forensic samples, population structure, endogamy, database composition, and genealogical record availability. Ethical and regulatory issues involving consent, privacy, database governance, law-enforcement access, data retention, and non-consenting relatives are considered. Overall, SNP-based forensic genomics can generate powerful investigative leads, but its outputs must be interpreted within method-specific analytical and evidentiary boundaries.

## 1. Introduction

Since the early 1990s, forensic genetics has relied heavily on DNA polymorphisms—commonly referred to as molecular markers—for individual identification, kinship testing, and the resolution of criminal and humanitarian cases [1,2]. Among these markers, short tandem repeats (STRs) became the cornerstone of human identification (HID) and gold standard in forensic genetics [3,4]. Their widespread forensic adoption is largely attributable to their broad genomic distribution [5,6], predominance in non-coding regions [6], and high levels of polymorphism, with heterozygosity often exceeding 70%, thereby providing strong discriminatory power for individual identification [5,6,7].

Despite their success, STR markers present several intrinsic limitations. Polymerase Chain Reaction (PCR)-based STR typing is susceptible to stochastic artifacts, including stutter peaks, allelic imbalance, and allele drop-out, particularly in low-template or degraded DNA samples [4]. In addition, the relatively large amplicon sizes required for conventional STR assays can compromise analytical performance when DNA is highly fragmented. These limitations have motivated the development and forensic implementation of alternative genetic marker systems [4,8].

**Figure 1:**
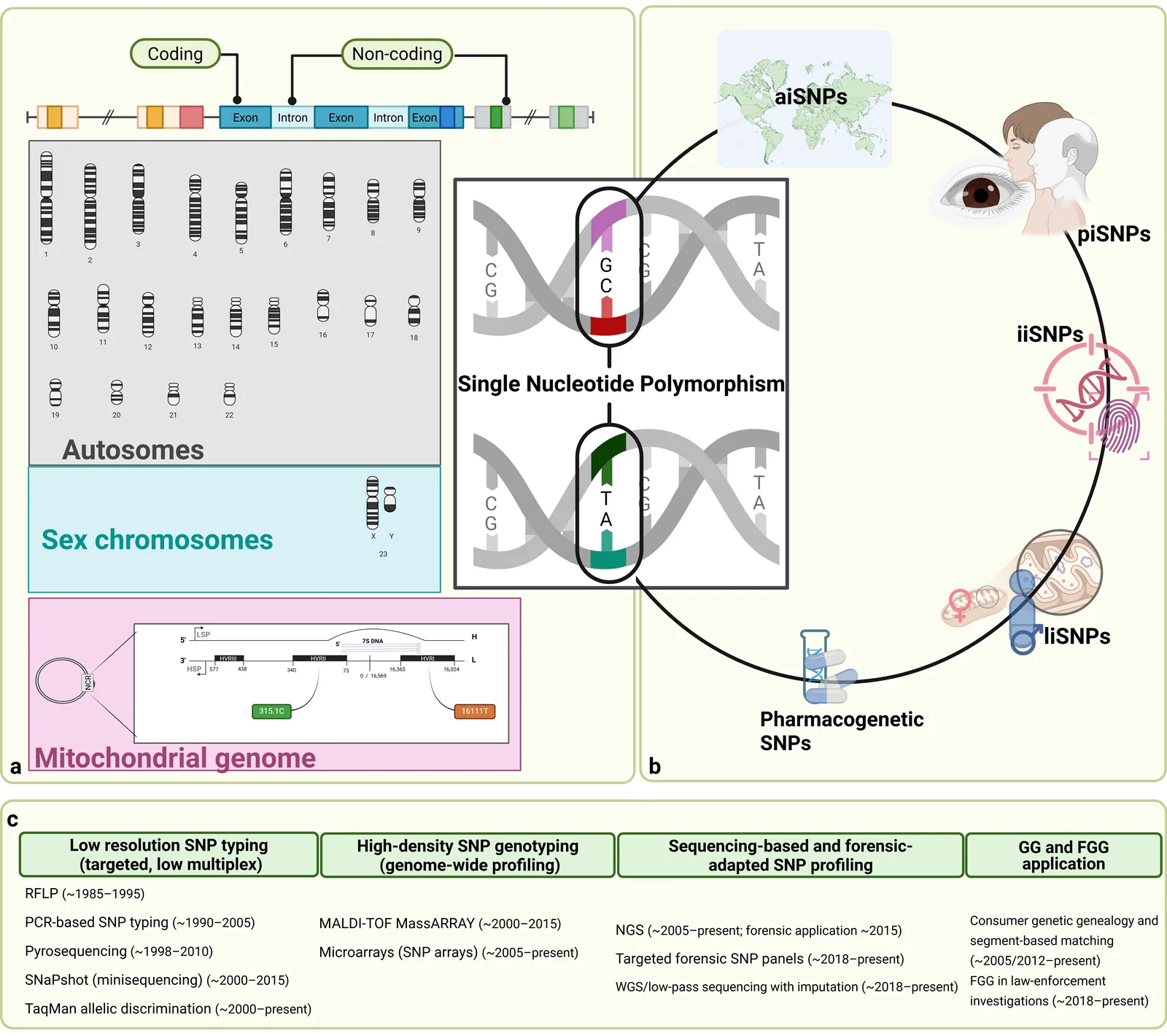
Overview of the genomic distribution, forensic classification, and technological evolution of SNPs. (**a**) SNPs occur throughout coding and non-coding regions of autosomal, sex-chromosomal, and mitochondrial DNA, supporting their use as genome-wide forensic markers. (**b**) According to forensic utility, SNPs can be classified as identity-informative (iiSNPs), ancestry-informative (aiSNPs), phenotype-informative (piSNPs), lineage-informative (liSNPs), and pharmacogenetic SNPs. (**c**) SNP-genotyping technologies have evolved from targeted assays to high-density genome-wide and sequencing-based approaches, enabling identity-by-descent segment detection, genetic genealogy database matching, and forensic genetic genealogy applications for distant kinship inference [19,20,21,22,23,24]. Created in BioRender. Becerra, D. (2026) https://BioRender.com/f7zbpn1.

Among these alternatives, Single-Nucleotide Polymorphisms (SNPs) have emerged as a powerful complementary—and, in some contexts, alternative—class of markers. SNPs consist of single-base substitutions in the DNA sequence [4,8] and represent the most abundant form of human genetic variation, occurring approximately once every 1000 base pairs (bp) throughout the genome [9,10]. They are distributed across coding and non-coding regions of the autosomal, sex-chromosomal, and mitochondrial genomes (Figure 1a) [9,10].

In forensic genetics, SNPs are commonly classified according to their principal forensic utility in individual identification, lineage analysis, biogeographic-ancestry inference, and prediction of externally visible characteristics (Figure 1b) [10,11,12,13]. Identity-informative SNPs (iiSNPs) are primarily used for individual identification and differentiation and may be located on autosomes or sex chromosomes [10]. Lineage-informative SNPs (liSNPs), located in mitochondrial DNA (mtDNA) and the Y chromosome, are useful for evolutionary analyses and kinship investigations involving distant relationships, including missing-person identification across multiple generations [10,11]. Ancestry-informative SNPs (aiSNPs) enable inference of biogeographic ancestry [10,12]. Phenotype-informative SNPs (piSNPs), typically located in coding or regulatory regions, are used to predict externally visible characteristics such as eye, hair, and skin pigmentation, as well as selected morphological traits [3,10,13]. In addition, pharmacogenetic SNPs have emerged within molecular forensic pathology and are increasingly applied in post-mortem “molecular autopsies” to investigate genetically mediated causes of death [10].

Several characteristics make SNPs particularly attractive for forensic applications [10]. First, their short amplicon requirements (typically 50–150 bp) facilitate successful amplification of highly degraded DNA fragments [8]. Second, SNPs exhibit substantially lower mutation rates than STRs (~10^−8^ vs. ~10^−3^ per generation) [1,14], increasing marker stability in kinship analyses across multiple generations [9,15]. Third, SNP genotyping is highly compatible with massively parallel sequencing (MPS) technologies, enabling the simultaneous analysis of hundreds to millions of loci within a single assay [8,16,17]. Although individual SNPs are typically biallelic and therefore less informative per locus than STRs, their high genomic density enables the interrogation of large marker panels, substantially increasing cumulative statistical power [8,18]. This technological progression from targeted SNP typing to high-density genome-wide genotyping and sequencing-based forensic profiling is summarized in Figure 1c [19,20,21,22,23,24].

Beyond their role in individual identification, genome-wide SNP data enable the detection of chromosomal segments shared identically by descent (IBD), providing a robust framework for kinship inference across multiple generations [25,26]. These developments have gained relevance with the emergence of forensic genetic genealogy (FGG), in which SNP profiles derived from unknown forensic samples are compared against large genetic genealogy databases to identify potential relatives and generate investigative leads [23,27,28]. Given the rapid methodological, computational, and ethical evolution of this field, a comprehensive synthesis of SNP-based approaches for distant kinship inference is warranted [23,28]. This narrative review was based on peer-reviewed literature and selected authoritative institutional or policy sources addressing SNP-based distant kinship inference, IBD detection, genetic genealogy database matching, FGG, targeted forensic SNP panels, low-coverage sequencing, and forensic kinship likelihood-ratio frameworks. Priority was given to peer-reviewed studies, validation papers, case reports, and policy or regulatory documents directly relevant to forensic implementation. Accordingly, this review examines SNP-based distant kinship inference as a broader forensic-genomic framework and distinguishes among three related but non-equivalent analytical contexts: general statistical and computational methods for modeling genomic relatedness, segment-based matching approaches used in genetic genealogy databases, and forensic SNP-based kinship methods applied outside genetic genealogy. Unlike previous reviews focused primarily on FGG practice, case profiles, ethical implications, or SNP technologies, the added value of this review lies in integrating the biological basis of IBD sharing, SNP marker-density requirements, statistical and computational relationship-inference frameworks, and the operational transition to FGG workflows, while clarifying the conceptual boundaries among genetic genealogy database matching, formal forensic kinship testing, and other SNP-based relationship inference approaches. Within this framework, FGG is discussed as a major operational application in which genome-wide SNP matches generate investigative leads requiring genealogical reconstruction and independent forensic confirmation.

## 2. Terminology and Scope

Terminology in this field remains heterogeneous. The terms forensic genetic genealogy (FGG), investigative genetic genealogy (IGG), and forensic investigative genetic genealogy (FIGG) are commonly applied to highly overlapping practices that integrate genome-wide SNP analysis, genetic genealogy database matching, genealogical research, and investigative follow-up. In this review, FGG is adopted as the primary term, whereas IGG and FIGG are recognized as related designations used across different scientific, legal, and professional contexts. These terms should therefore be understood primarily as heterogeneous terminology rather than strictly distinct technical methodologies.

The main conceptual distinction is not between FGG, IGG, and FIGG themselves, but between SNP-based genetic genealogy lead generation, conventional STR-based familial searching, formal forensic kinship testing, and independent forensic confirmation. Traditional familial searching generally relies on STR profiles and is optimized for the detection of close relatives, whereas FGG is based on genome-wide SNP profiles, shared-segment detection, genetic genealogy database matching, and extended pedigree reconstruction [28,29,30,31,32]. Formal forensic kinship testing, in contrast, evaluates explicit relationship hypotheses using reference samples and statistical interpretation. Maintaining these boundaries avoids treating overlapping terminology as separate technical categories and helps distinguish investigative lead generation from evidentiary identification.

## 3. Genetic Basis of Distant Kinship Inference

Genome-wide SNP interrogation has transformed kinship inference by enabling the detection of chromosomal segments shared IBD. These segments are inherited from common ancestors and progressively fragmented through recombination across successive meioses. Consequently, the amount, number, and length of shared IBD segments provide a genomic framework for estimating biological relatedness across multiple degrees of separation, extending analytical capabilities beyond conventional STR-based kinship testing [1,14,25,26,28,31,33].

This segment-based framework differs fundamentally from traditional STR-based comparisons, which rely on a limited number of largely independent loci. In contrast, dense SNP datasets reconstruct patterns of shared genomic segments across chromosomes while incorporating genetic linkage, recombination, marker density, and genotype quality [25,26,33,34,35]. Within this context, the distinction between identity-by-state (IBS) and IBD is critical: IBS refers to observed allele sharing, whereas IBD implies inheritance from a recent common ancestor and therefore requires interpretation within an appropriate population and genealogical framework [25,26,34].

**Figure 2:**
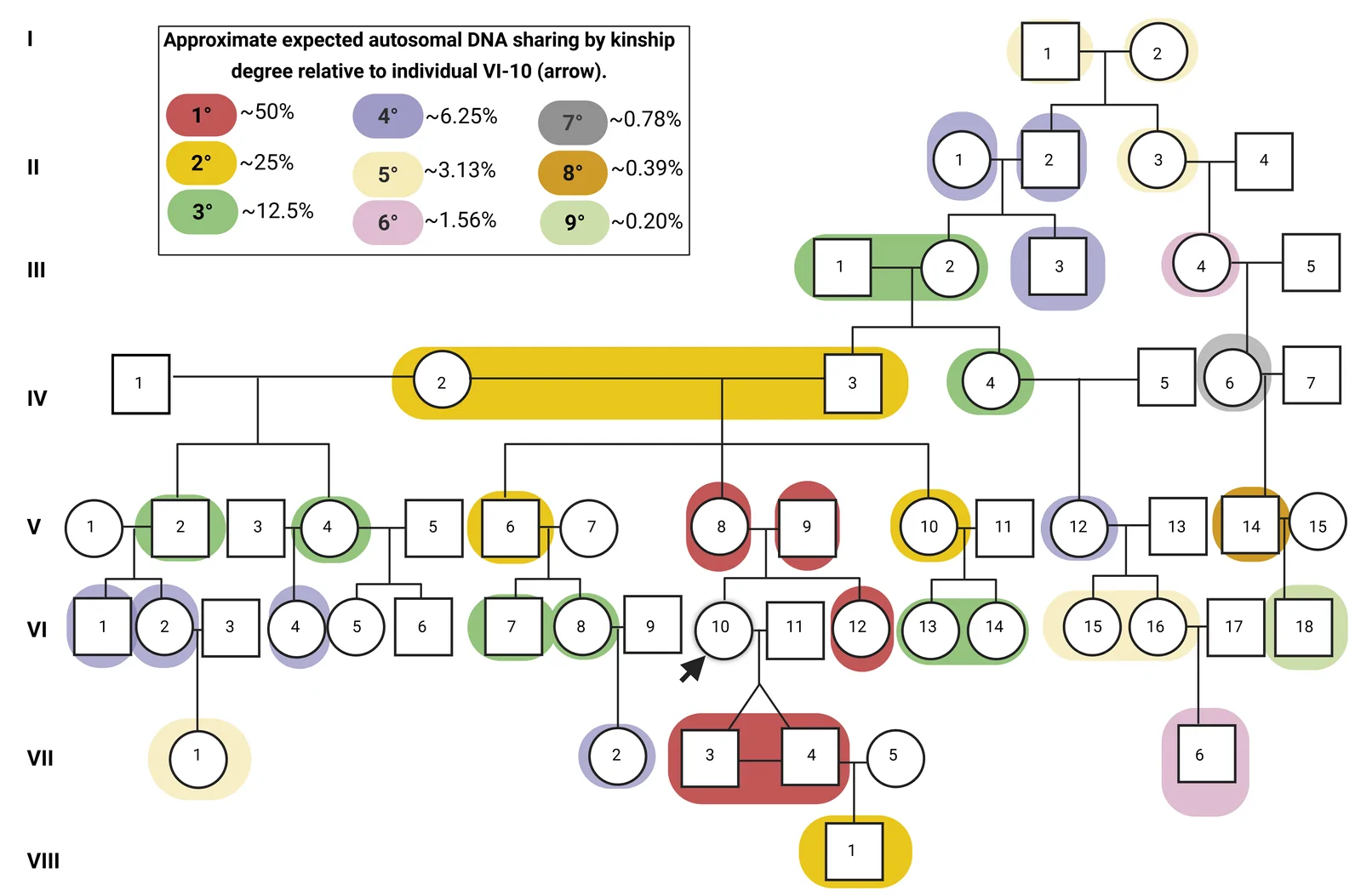
Expected autosomal DNA sharing across first- to ninth-degree relationships relative to the proband VI-10 (black arrow). The schematic illustrates the progressive decline in shared autosomal DNA and the fragmentation of inherited IBD segments with increasing genealogical distance. Roman numerals indicate generations. Individuals VII-3 and VII-4 are monozygotic twins; the horizontal line connecting their twin branches denotes monozygosity and does not represent a mating relationship. Created in BioRender. Becerra, D. (2026) https://BioRender.com/1olkp0u.

The expected proportion of autosomal DNA shared between relatives decreases with each meiosis separating them from a common ancestor (Figure 2). Close relatives generally share larger genomic proportions in longer chromosomal segments, whereas distant relatives share progressively fewer and shorter IBD tracts [25,26,33,36]. However, realized genomic sharing may deviate substantially from theoretical expectations because recombination, chromosomal segregation, crossover interference, and sex-specific recombination patterns introduce variability in both the total amount of shared DNA and the distribution of IBD segment lengths [36,37]. Consequently, adjacent relationship categories may exhibit overlapping distributions of shared DNA, particularly as genealogical distance increases [33,38,39].

In genetic genealogy, shared autosomal DNA is commonly summarized in centiMorgans (cM), a unit of genetic distance reflecting recombination probability. Approximately 1 cM corresponds to a 1% probability of recombination between two loci during a single meiosis. Total shared cM, together with the number of shared segments and maximum segment length, provides an empirical indicator of relationship proximity. However, these metrics remain probabilistic rather than definitive, and shared-cM ranges may overlap substantially across relationship categories [25,26,33,38,39,40,41,42].

As genetic distance increases, a genealogical–genetic tradeoff becomes apparent: the number of potential genealogical relatives expands with each generation, whereas the probability of detecting informative autosomal IBD sharing progressively decreases [25,26,38]. Some distant relatives may retain detectable IBD segments, whereas others may share little or no detectable autosomal DNA depending on recombination history, marker density, genotyping quality, and analytical sensitivity [25,26,37,38,43,44]. This stochasticity represents a fundamental limitation of distant kinship inference and explains why genetic genealogy matches should be interpreted as probabilistic indicators of relatedness rather than direct evidence of a specific relationship category [28,29,31,38].

At a population-genomic scale, ancestry-specific IBD sharing and ancestry-tract-length distributions have also been used to infer the source and timing of historical admixture. Ioannidis et al. [45] applied these approaches to support pre-European Native American gene flow into eastern Polynesia. Although this differs from forensic pedigree analysis in inferential scale and purpose, it illustrates how recombination progressively shortens inherited tracts and thereby preserves temporal information about shared ancestry.

Marker density and data quality are therefore critical determinants of performance. Segment-based IBD detection for genetic genealogy database matching generally requires dense genome-wide SNP profiles comprising hundreds of thousands of markers to provide sufficient sensitivity for distant relationships (Figure 1c) [23,28,31,38,42,44,46]. Lower-density forensic SNP panels may support targeted kinship testing within defined analytical frameworks, particularly for closer or moderately distant relationships, but they should not be considered equivalent to high-density genetic genealogy database searches [24,38,43,47,48]. This distinction is especially relevant in forensic contexts, where evidentiary samples may be degraded, low-template, inhibited, contaminated, mixed, or incomplete, thereby reducing SNP recovery, increasing genotyping error, fragmenting true IBD segments, and decreasing the sensitivity of segment-based matching [24,28,38,44].

## 4. SNP-Based Approaches for Distant Kinship Inference

The biological principles underlying identity-by-descent (IBD) sharing described in the previous section provide the foundation for several analytical strategies used to infer distant genetic relationships. However, these approaches differ substantially in their objectives, statistical frameworks, marker-density requirements, and forensic applications. In forensic genetic genealogy (FGG), the initial analytical step typically consists of segment-based matching against genetic genealogy databases to identify potential biological relatives. In contrast, formal forensic kinship testing evaluates predefined relationship hypotheses using likelihood-based, pedigree-based, genotype-likelihood, or segment-based statistical models. Additional SNP-based methodologies—including targeted capture panels, forensic SNP panels, low-coverage sequencing strategies, Bayesian inference, and machine-learning approaches—also contribute to relationship inference but should not be regarded as interchangeable with routine genetic genealogy database matching. Distinguishing among these analytical frameworks is essential because they differ in their assumptions, statistical interpretation, forensic validation, and operational objectives [25,26,28,29,31,33,34,38].

### 4.1. Segment-Based Matching in Genetic Genealogy Databases

Genetic genealogy (GG) databases identify potential biological relatives through segment-based comparison of genome-wide autosomal SNP profiles. Publicly described matching frameworks compare pairs of individuals across the autosomes to detect long chromosomal segments that are likely to be identical by descent (IBD). Detected matches are typically summarized using total shared centiMorgans (cM), the number of shared segments, the length of the longest shared segment, and an estimated relationship range [25,26,29,31,33,38,40,41,42,46]. Importantly, these outputs should not be interpreted as forensic likelihood ratios (LRs). Instead, they represent genetic genealogy match indicators that support genealogical triangulation, documentary research, and pedigree reconstruction [28,29,30,31,49].

This analytical strategy differs fundamentally from formal forensic kinship testing. Rather than evaluating a predefined relationship hypothesis, GG database searches compare an unknown SNP profile against large collections of voluntarily contributed genetic profiles to identify potential relatives. Relationship estimates are generally derived from empirical shared-segment metrics together with platform-specific algorithms, many of which remain proprietary or only partially described. Consequently, a genetic genealogy match constitutes an investigative lead rather than a forensic identification and must subsequently be evaluated through genealogical reconstruction, demographic and historical information, investigative follow-up, and independent forensic confirmation [28,29,30,31,49].

As discussed in Section 3, the sensitivity of this approach depends strongly on marker density and genotype quality. Accordingly, the output generated by GG databases should be interpreted as evidence of potential biological relatedness rather than proof of identity. Its principal forensic value lies in identifying relatives who may facilitate reconstruction of ancestral lineages and guide subsequent investigative and forensic analyses.

### 4.2. Identity-by-Descent Sharing and Pedigree Expectations

The analytical approaches described in the following sections are founded on the biological principles of Mendelian inheritance and identity-by-descent (IBD) sharing. As discussed in Section 3, genomic segments inherited from a common ancestor are progressively fragmented by meiotic recombination, resulting in shorter and fewer detectable IBD segments as genealogical distance increases. Consequently, distant kinship inference relies on probabilistic rather than deterministic patterns of genomic sharing [25,26,29,31,33,36,38].

A fundamental concept underlying pedigree-based relationship inference is the coefficient of relationship (*r*), which represents the expected proportion of the genome shared IBD between two individuals through a given genealogical pathway. If *k* denotes the number of meioses separating two individuals along a single transmission path, the contribution of that path is given by:

$$r=\sum (1/2{)}^{k}$$

where the summation accounts for all independent inheritance pathways connecting the two individuals within a pedigree [25,26].

This formulation reflects the principle that each meiosis transmits approximately one-half of the parental genome to the offspring. Consequently, first-degree relatives are expected to share approximately 50% of their autosomal genome despite differing pedigree structures. For example, parent–offspring pairs are connected through a single meiotic transmission, whereas full siblings share two independent parental inheritance pathways that together produce the same expected coefficient of relationship [25,26].

Although the coefficient of relationship provides the theoretical expectation of genomic sharing, the realized amount of shared DNA varies among individuals because recombination occurs stochastically. As a result, close relatives generally share multiple long IBD segments, whereas distant relatives tend to share fewer and shorter segments distributed across the genome. Moreover, the distributions of total shared DNA, segment number, and segment length increasingly overlap with genealogical distance, limiting the precision of relationship classification based solely on empirical sharing metrics [25,29,31,33,38,39,40,41,42].

Modern computational methods therefore estimate relatedness by integrating observed genomic sharing with probabilistic models of inheritance rather than relying exclusively on empirical shared-DNA thresholds. In genome-wide datasets, IBD detection is commonly performed using hidden Markov models (HMMs), which jointly model recombination patterns, allele frequencies, marker density, and genotyping uncertainty to infer the most likely distribution of IBD segments across the genome [25,29,33,38,44]. These probabilistic approaches improve the detection of short shared segments but remain sensitive to population structure, endogamy, and genotype quality, particularly when evaluating distant relationships [29,37,38,46].

Accordingly, the coefficient of relationship should be regarded as the theoretical foundation of pedigree-based kinship inference, whereas the observed genomic sharing detected in modern SNP datasets represents a stochastic realization of that expectation. This distinction underlies the statistical frameworks discussed in the following sections and explains why relationship inference requires explicit probabilistic modeling rather than simple comparison of shared DNA values.

### 4.3. Likelihood-Based Frameworks for SNP-Based Kinship Inference

Formal forensic kinship analysis differs fundamentally from genetic genealogy database matching because it evaluates predefined relationship hypotheses rather than performing exploratory searches across large collections of genetic profiles. Consequently, evidentiary interpretation relies on statistical models that quantify the probability of the observed genetic data under competing pedigree hypotheses rather than on empirical measures of shared DNA alone [25,26,29,31,33,34,38].

The principal statistical framework used in forensic kinship analysis is the likelihood ratio (LR), which compares the probability of observing the genetic evidence under two competing hypotheses. For example, one hypothesis may represent a specified biological relationship, whereas the alternative assumes that the individuals are unrelated. The LR is defined as:

$$LR=\frac{P(G|H_{1})}{P(G|H_{2})}$$

where *G* denotes the observed multilocus genotype data.

For genome-wide SNP datasets, likelihood calculations are considerably more complex than those used for conventional STR panels because dense SNP markers cannot generally be considered independent. Instead, analytical models must account for allele frequencies, linkage disequilibrium (LD), recombination, and genotyping uncertainty when estimating the probability of the observed data [25,26,33,34,38].

Several complementary analytical strategies have therefore been developed. Some methods reduce marker dependence through marker selection or LD pruning when approximate locus independence is assumed. Others explicitly incorporate chromosomal linkage and recombination into pedigree-based or segment-based models that evaluate the probability of observing the detected IBD sharing under alternative relationship hypotheses [25,26,33,34,38]. These approaches are particularly important for genome-wide datasets because recombination patterns contain information that contributes directly to relationship inference.

Genome-wide IBD detection is commonly implemented using hidden Markov models (HMMs), which estimate the most likely sequence of IBD and non-IBD states along the genome while accounting for recombination, allele frequencies, marker density, and genotyping uncertainty [25,29,33]. These probabilistic models substantially improve the detection of shared chromosomal segments, particularly shorter IBD tracts that are informative for more distant relationships, although their performance remains influenced by marker density, genotype quality, and population characteristics [33,38,44].

Bayesian inference represents a complementary probabilistic framework in which genotype data are combined with prior information regarding pedigree structure or other model parameters to estimate the posterior probability of competing relationship hypotheses. Hierarchical Bayesian models may additionally incorporate uncertainty in allele frequencies, recombination rates, mutation processes, and population structure, providing a flexible framework for modeling complex genealogical scenarios [25,29,31,38,50,51]. Although these approaches are computationally demanding and remain less common in routine forensic casework than likelihood-based methods, they have become increasingly relevant for uncertainty modeling and methodological research in genome-wide kinship inference [29,38,51].

Accurate modeling of genotyping uncertainty is particularly important in forensic applications because degraded, low-template, inhibited, contaminated, or incomplete samples may accumulate small genotyping errors across thousands of SNPs, potentially influencing relationship inference [24,28,38,44]. Consequently, robust likelihood-based analyses require appropriate consideration of marker dependence, population structure, genotype quality, and analytical uncertainty when evaluating genome-wide evidence [29,36,38,52,53,54].

Unlike formal forensic kinship testing, genetic genealogy database searches do not typically generate likelihood ratios for predefined pedigree hypotheses. Instead, they identify potential relatives through shared-segment matching, after which formal statistical evaluation may be performed if suitable reference samples become available. Accordingly, likelihood-based analysis should be viewed as a complementary forensic framework that supports evidentiary evaluation following the generation of investigative leads through genetic genealogy [28,29,31,54].

### 4.4. Forensic SNP-Based Kinship Methods Beyond Genetic Genealogy Databases

Not all forensic SNP-based kinship inference relies on genetic genealogy (GG) databases. Several analytical approaches have been developed for forensic scenarios involving predefined reference samples, historical remains, missing-person investigations, low-coverage sequencing data, or direct candidate comparisons rather than open-ended database searches. Although these methodologies also exploit genome-wide SNP information, they operate within controlled forensic comparison frameworks and should therefore be distinguished from the investigative workflow of forensic genetic genealogy (FGG) [24,36,38,47,48,52,53].

One important application is targeted SNP capture combined with massively parallel sequencing (MPS), which enables the recovery of informative nuclear SNPs from highly degraded skeletal or historical remains. Gorden et al. [55], for example, demonstrated that targeted SNP capture can support extended kinship analysis even when DNA quality is insufficient for conventional genome-wide microarray genotyping. Such approaches have expanded the applicability of SNP-based kinship inference to forensic contexts in which highly fragmented DNA would otherwise preclude genome-wide analysis.

Similarly, low-coverage sequencing strategies have broadened the range of forensic samples suitable for relationship inference. Nguyen et al. [56] developed IBDGem, a computational framework designed to detect IBD sharing from low-coverage shotgun sequencing data, thereby facilitating kinship estimation when complete, high-quality genotype profiles cannot be obtained [56].

Targeted forensic SNP panels represent another important analytical strategy. Systems such as the ForenSeq™ Kintelligence assay, together with related computational approaches, provide optimized marker sets for forensic kinship analysis in defined casework scenarios, particularly for close and moderately distant relationships [24,43,47,48]. Although these panels require substantially fewer markers than consumer genome-wide SNP arrays, they have been specifically designed to maximize kinship information from forensic samples while maintaining compatibility with degraded DNA [24,38,43,47,48]. Nevertheless, their marker density and analytical objectives differ from those of high-density genetic genealogy database searches, and they should therefore be regarded as complementary forensic tools rather than interchangeable substitutes for GG database matching.

Collectively, these approaches illustrate that SNP-based distant kinship inference extends well beyond forensic genetic genealogy. In addition to database-assisted investigations, genome-wide SNP analyses increasingly support historical identifications, missing-person investigations, targeted kinship testing, and other forensic applications in which relationship hypotheses are evaluated within controlled analytical frameworks rather than through exploratory genealogy database searches.

### 4.5. Emerging Computational Approaches

Recent advances in artificial intelligence have introduced machine-learning approaches as complementary tools for SNP-based kinship inference. Rather than explicitly modeling inheritance probabilities, these methods learn patterns of genomic similarity from training datasets and use multiple genomic features—including shared DNA, IBD segment characteristics, allele-sharing statistics, and population-genetic information—to predict relationship categories [57,58].

Algorithms such as random forests, gradient-boosting methods, and neural networks have shown promising performance for distinguishing distant relationships by capturing complex interactions among genomic variables that may not be fully represented by conventional statistical models [57,58]. These approaches may therefore improve relationship classification, particularly when genomic sharing falls within overlapping ranges characteristic of more distant relatives.

Despite these advances, machine-learning methods remain complementary to established forensic statistical frameworks. Unlike likelihood ratios, which provide formally interpretable measures of evidentiary strength, many machine-learning models function primarily as predictive classifiers whose outputs may be difficult to translate into legally interpretable evidence. Consequently, their current value lies in supporting relationship prioritization and methodological development rather than replacing formal forensic hypothesis testing or genetic genealogy database matching [59].

### 4.6. Analytical Limitations and Sources of Uncertainty

Despite the considerable advances enabled by genome-wide SNP analysis, distant kinship inference remains subject to several biological, statistical, and technical limitations that influence the interpretation of relatedness estimates. These limitations affect both formal forensic kinship testing and genetic genealogy database matching, although their impact differs according to the analytical framework employed [28,29,38,39,44].

One of the principal sources of uncertainty arises from the stochastic nature of meiotic recombination. As genealogical distance increases, the realized proportion of shared DNA varies substantially among relatives belonging to the same pedigree class, resulting in broad and overlapping distributions of shared genomic segments. Consequently, increasingly distant relationships become progressively more difficult to distinguish using shared DNA metrics alone [25,26,29,33,36,37,38,39].

Population history further complicates relationship inference. Population structure, founder effects, endogamy, and pedigree collapse may increase background levels of genomic sharing or create multiple inheritance pathways between individuals, producing greater IBD sharing than predicted by simplified pedigree models. In formal forensic analyses, these factors can be partially addressed through the use of population-specific allele frequencies and appropriate statistical modeling. In contrast, genetic genealogy database matching relies primarily on genealogical interpretation to recognize such population-specific patterns and avoid overestimating biological relatedness [29,37,38,46,60].

Analytical performance is also strongly influenced by DNA quality and data completeness. Degraded or low-template forensic samples, low sequencing coverage, genotyping errors, and phasing inaccuracies may reduce the detection of true IBD segments or alter their estimated boundaries, thereby decreasing sensitivity for distant relationship inference [24,28,38,39,44,55,56,61,62]. Likewise, harmonization among different SNP platforms, genotype-calling strategies, and bioinformatic pipelines remains an important consideration when integrating data generated using different technologies [28,29,38,39,44].

Finally, genome-wide analyses inherently involve the accumulation of uncertainty across thousands or hundreds of thousands of markers. Small systematic biases associated with allele frequency estimation, recombination maps, genotype calling, imputation, or marker selection may collectively influence likelihood ratios, posterior probabilities, or relationship classification. Accordingly, relatedness estimates should always be interpreted within the assumptions, validation limits, and intended application of the analytical framework from which they were generated [28,29,38,39,44].

Recognizing these limitations is essential for the appropriate interpretation of SNP-based kinship inference. Whether applied to formal forensic comparisons or forensic genetic genealogy, genomic relatedness estimates should be regarded as probabilistic evidence that must be evaluated together with genealogical, investigative, and independent forensic information. This integrated perspective provides the foundation for the operational FGG workflow described in the following section.

## 5. From Distant Kinship Inference to Forensic Genetic Genealogy

**Figure 3:**
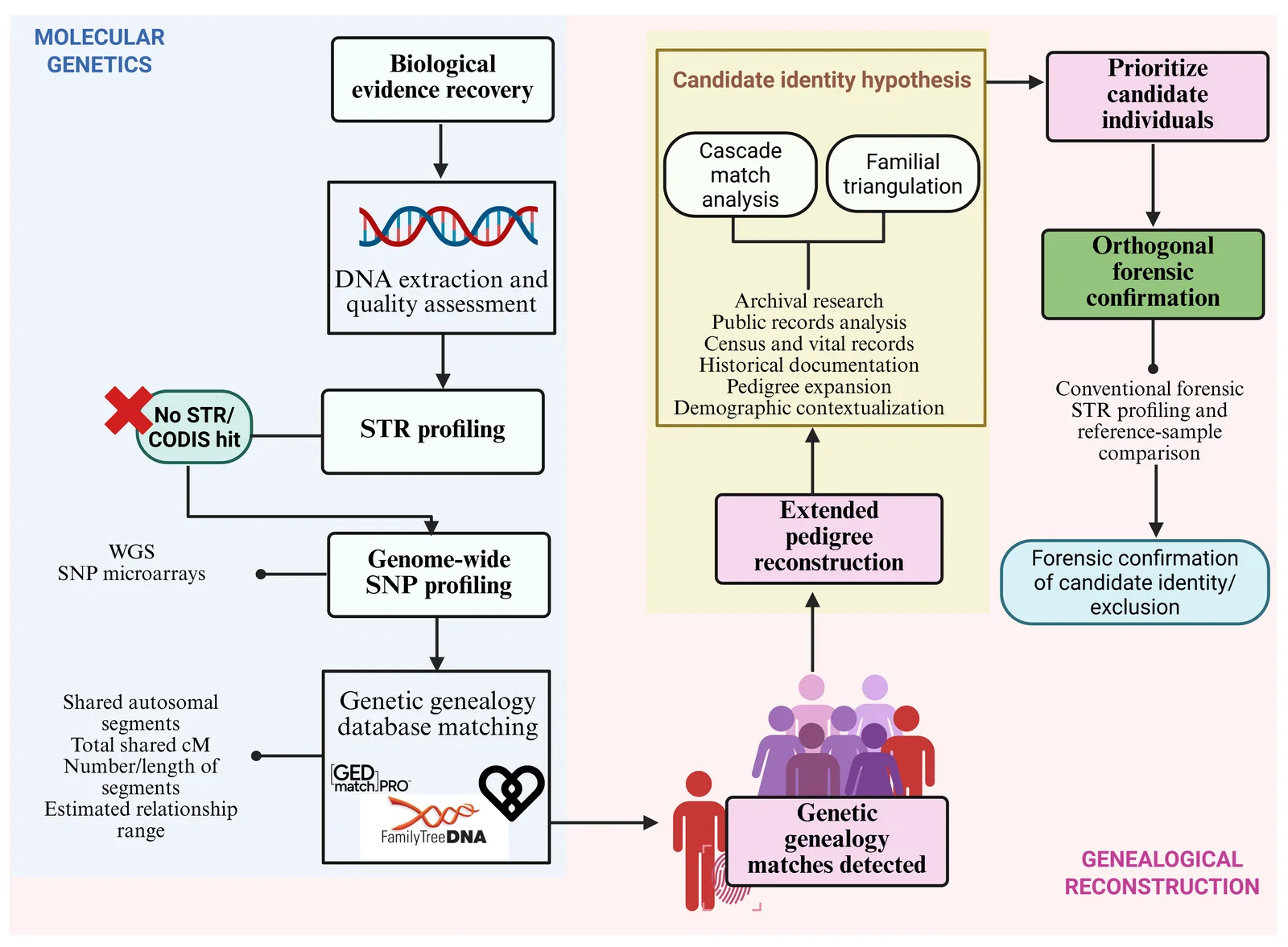
Operational workflow of FGG. The workflow begins with biological evidence recovery, DNA extraction, quality assessment, and conventional STR profiling. When no STR/CODIS hit or conventional investigative lead is obtained, genome-wide SNP profiling—using whole-genome sequencing [WGS], SNP microarrays, or validated forensic SNP workflow—may be used for authorized GG database matching. Genetic matches support genealogical reconstruction and candidate identity hypotheses, but final identification or exclusion requires independent forensic confirmation, typically through STR profiling and reference-sample comparison [27,28,29,30,31,44,46,49]. Created in BioRender. Becerra, D. (2026) https://BioRender.com/h8bchs5.

Transitioning from SNP-based distant kinship inference to FGG requires translating genetic relatedness signals into an operational investigative workflow integrating genome-wide SNP profiling, genetic genealogy database searches, genealogical reconstruction, and independent forensic confirmation (Figure 3).

At the laboratory level, FGG generally begins with the same evidentiary material analyzed in conventional forensic DNA casework. Biological evidence is recovered, DNA is extracted and quantified, and STR profiling is typically performed first to search national or regional forensic databases such as the Combined DNA Index System (CODIS), or equivalent systems where applicable [28,54,63,64]. If no direct STR match or conventional investigative lead is obtained, genome-wide SNP profiling may subsequently be pursued using high-density SNP microarrays, whole-genome sequencing, or validated forensic SNP workflows, depending on sample quality, case requirements, and database compatibility [17,18,23,24,28,31,38,44]. The selected profiling workflow must satisfy the profile-completeness, marker-density, and database-compatibility requirements summarized in Section 3 and Table 1.

Once uploaded or compared within an authorized genetic genealogy environment, the SNP profile is evaluated against database participants who have consented—according to the platform’s applicable policies—to forensic or law-enforcement matching. The resulting match list does not provide a final identification; instead, it generates a ranked set of potential biological relatives whose shared segments may indicate common ancestry [27,28,29,30,31,49]. The strength and utility of these matches depend on the amount of shared DNA, number and length of shared segments, presence of endogamy or background relatedness, representativeness of the database, and availability of documentary genealogical records [28,29,38,49].

Once candidate genetic matches are identified, the investigation transitions from molecular genetics to genealogical reconstruction. This stage integrates archival research, public and vital records, obituaries, census documents, demographic contextualization, historical documentation, and pedigree expansion to identify shared ancestors and reconstruct extended family networks [28,29,30,49]. Key strategies include familial triangulation and cascade searching, in which multiple genetic matches are evaluated jointly to identify overlapping lineages, common ancestral couples, and descendancy pathways potentially connecting the unknown forensic profile to a candidate individual or family branch [28,29,30,31,49].

Within this framework, genealogical reconstruction functions primarily as a mechanism for hypothesis generation. Candidate identities are progressively refined using demographic and investigative filters, including age, sex, geographic location, migration history, temporal plausibility, family structure, and case-specific investigative information [28,29,30,49]. When necessary, targeted testing of additional relatives may further refine pedigree reconstruction or evaluate competing hypotheses, although such testing should be distinguished from the initial GG database match [28,29,49].

Final identification in FGG workflows requires independent forensic confirmation, commonly through STR profiling, direct comparison with a reference sample, or another validated forensic identification procedure [28,29,44]. Accordingly, the operational boundary between genetic genealogy lead generation and forensic confirmation should remain explicit in both casework practice and evidentiary interpretation. Because FGG involves voluntarily contributed genealogical data and may affect biological relatives who did not directly participate in testing, implementation also requires privacy safeguards, informed consent, database governance, law-enforcement access policies, and jurisdiction-specific oversight frameworks [29,30,49,54,63,65,66,67,68,69,70,71,72].

**Table 1:** Representative publicly reported FGG milestones in criminal investigations and unidentified human remains identification. Cases were included when publicly available sources reported the use of FGG involving genome-wide SNP-based genetic genealogy database searching for suspect identification or UHR resolution. This table is representative rather than exhaustive; for a full list of publicly reported cases, see Dowdeswell [32].

A. Criminal Investigations Resolved or Advanced Using FGG					
Suspect/Case	Country	Incident Year	Resolution Year	Lead-Generating Entity, If Publicly Reported	Significance
Joseph James De Angelo/Golden State Killer	USA	1974–1986	2018	Barbara Rae-Venter/law-enforcement collaboration	First widely recognized law-enforcement use of SNP-based FGG [23,30,31,73].
William Earl Talbott II/Cook–Van Cuylenborg murders	USA	1987	2018	Parabon NanoLabs/genetic genealogy collaboration	Early demonstration of FGG scalability in a cold-case homicide [30,32].
Raymond Charles Rowe/Christy Mirack murder	USA	1992	2018	Parabon NanoLabs/law enforcement	FGG lead followed by independent forensic confirmation [30,32].
Roy Charles Waller/NorCal Rapist	USA	1991–2006	2018	Law enforcement/genetic genealogy collaboration	Serial sexual-assault investigation advanced through FGG [32,74].
Brian Leigh Dripps/Angie Dodge murder	USA	1996	2019	Parabon NanoLabs/Idaho Falls Police Department	First reported FGG-enabled exoneration [30,32].
Daniel Nyqvist/Linköping double murder	Sweden	2004	2020	Swedish police	First confirmed European FGG case [75].
**B. Identification of Unidentified Human Remains Using FGG**					
**Case Name**	**Country**	**Found Year**	**Identification Year**	**Lead-Generating Entity, If Publicly Reported**	**Significance**
Bear Brook/Allenstown Four	USA	1985/2000	2017–2019	DNA Doe Project/Parabon involvement reported in public sources	Early complex UHR identification integrating genealogy and database matching [30,32,74].
Boy in the Box/Joseph Augustus Zarelli	USA	1957	2022	Not consistently specified in scientific literature	Long-unidentified child resolved through FGG [74,76].
Lady of the Dunes/Ruth Marie Terry	USA	1974	2022	Not consistently specified in scientific literature	Longstanding homicide victim identified through SNP-based genealogy [74,76].
Sandy Point Man/Christopher Luke Moore	Australia	2017	2023; case report published 2024	Victorian Institute of Forensic Medicine-led multidisciplinary collaboration; FGG work led by Runa Daniel according to the case report	Multidisciplinary Australian UHR identification integrating FGG and historical evidence [77].
Nogales John Doe/Donald Hadland Jr.	USA	2002	2023	Not consistently specified in scientific literature	Long-term UHR case resolved through FGG [74,76].
Rhinelander John Doe/Norman Grasser	USA	1980	2023	Not consistently specified in scientific literature	Missing-person identification through genealogical reconstruction [74,76].

FGG: forensic genetic genealogy; SNP: single nucleotide polymorphism; UHR: unidentified human remains.

## 6. Operational Applications of Forensic Genetic Genealogy

In 2018, the identification of the Golden State Killer suspect marked the first widely recognized law-enforcement application of FGG based on genome-wide SNP data and genetic genealogy database searching [23,30,31,73]. This milestone demonstrated that genetic genealogy matches, when integrated with documentary research and extended pedigree reconstruction, could generate investigative leads in cases where conventional STR database searches had failed to produce direct matches [28,30,31,49]. Since then, FGG has expanded across criminal investigations and unidentified human remains (UHR) casework and has consolidated as an emerging interdisciplinary forensic field, although its operational success remains strongly influenced by database accessibility, sample quality, SNP density, legal frameworks, and the availability of genealogical records [28,30,31,32,76,78].

**Figure 4:**
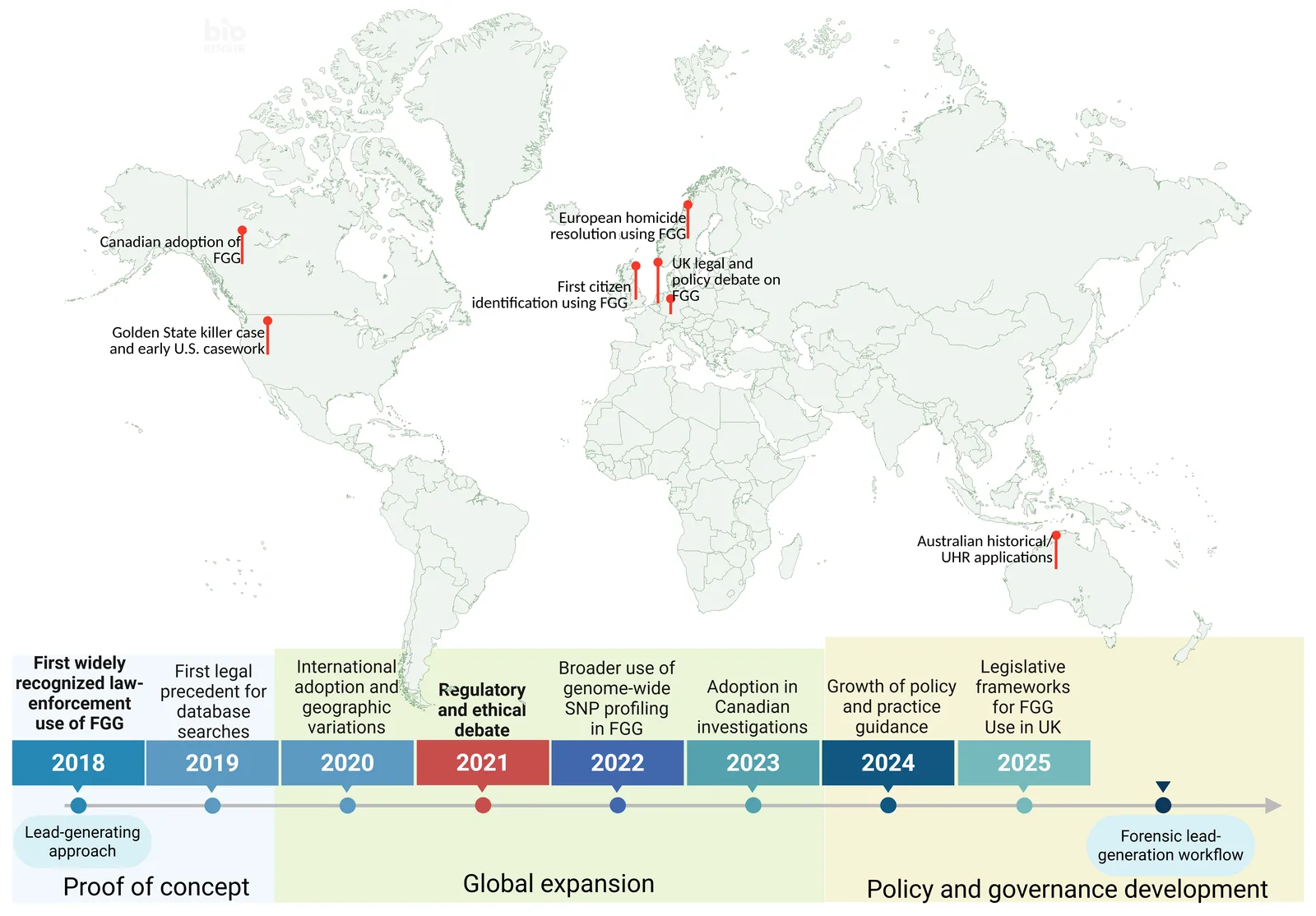
Selected milestones in the forensic implementation of genetic genealogy. The figure summarizes representative developments discussed in this review, including the 2018 Golden State Killer investigation, early legal and regulatory developments, international adoption of FGG, and the expansion of SNP-based workflows for criminal investigations and unidentified human remains casework. The timeline is representative rather than exhaustive [23,24,30,31,32,61,62,68,69,70,73,75,76,79,80,81]. Created in BioRender. Becerra, D. (2026) https://BioRender.com/b3gkggy.

Importantly, FGG should be distinguished from earlier STR-based familial searching approaches. Pre-FGG kinship-based strategies, including familial searches in forensic STR databases, demonstrated the investigative utility of indirect genetic matching but did not employ the genome-wide SNP-based genetic genealogy workflow that characterizes contemporary FGG [30,54,63,71]. Accordingly, pre-2018 STR familial-searching cases are discussed here as methodological precursors rather than operational FGG investigations and are therefore excluded from the revised FGG case table (Figure 4, Table 1). Table 1 lists representative milestone FGG cases; for a full list of publicly reported cases, see Dowdeswell [32].

### 6.1. Criminal Investigations

FGG has been used to generate investigative leads in both cold and active criminal investigations, particularly homicides and sexual assaults in which biological evidence is attributable to a single unknown contributor and conventional STR database searches have failed to identify a suspect [28,31,32,82,83]. In these investigations, a genome-wide SNP profile generated from crime-scene DNA is searched against genetic genealogy databases that permit forensic or law-enforcement use. The resulting genetic matches are interpreted as potential biological relatives, and genealogical reconstruction is subsequently used to identify common ancestors, reconstruct descent lines, and prioritize candidate individuals [28,29,31,49].

The Golden State Killer investigation served as the operational proof of concept for this approach, but subsequent investigations demonstrated that FGG could be applied beyond a single landmark case, including serial sexual assault investigations, cold-case homicides, and cross-jurisdictional investigations [30,31,32,73]. FGG may also contribute to the exclusion or correction of investigative assumptions when reconstructed pedigrees fail to support a candidate individual or when independent forensic confirmation excludes a proposed suspect [29,32]. For example, in the Angie Dodge homicide case, FGG was used to help identify crime-scene DNA that did not match the individual who had previously been convicted of the crime, resulting in the first FGG-based exoneration [30,32]. In criminal investigations, FGG functions as a lead-generation and hypothesis-development framework; final attribution requires independent forensic confirmation, typically through STR profiling or direct comparison with a reference sample [28,29,44].

### 6.2. Identification of Unidentified Human Remains

FGG has also emerged as an important tool in unidentified human remains (UHR) investigations when conventional missing-person database searches are negative. A major advantage of FGG in UHR casework is its ability to generate investigative leads even in the absence of direct family reference samples [61,62,76]. In these contexts, SNP profiles generated from skeletal or otherwise compromised remains may be searched against genetic genealogy databases to identify distant relatives, reconstruct family lineages, and generate identity hypotheses [61,62,76]. Greytak et al. [76] reviewed the laboratory, bioinformatic, and genealogical methodologies used in FGG for human remains identification and highlighted its growing role in UHR investigations.

Implementation in UHR investigations nevertheless remains highly dependent on DNA preservation and SNP profile completeness. Microarray-based FGG studies emphasize that sufficiently complete SNP profiles are necessary for reliable database searching, whereas compromised DNA may reduce SNP recovery, increase missing data rates, and decrease kinship-classification performance [61,62]. More recent forensic workflows using the ForenSeq™ Kintelligence system, targeted SNP panels, and capture-enrichment strategies further illustrate ongoing efforts to adapt SNP-based approaches to degraded or limited forensic material [24,62,79,80,81].

The Sandy Point case in Australia provides a useful example of why technically relevant and well-documented UHR investigations should be included in representative FGG milestone summaries. In this case, the absence of contextual information initially prevented the development of a viable identification hypothesis, and the eventual identification required integration of forensic anthropology, odontology, molecular biology, radiocarbon dating, historical investigation, and genealogical reconstruction [77]. The investigation ultimately resulted in the identification of Christopher Luke Moore and illustrates the multidisciplinary nature of UHR identification using FGG [77].

As in criminal investigations, genetic genealogy in UHR casework provides an investigative identity hypothesis that must subsequently be evaluated using historical records, missing-person information, biological profile consistency, DNA confirmation, and other independent lines of evidence [29,44,76]. This distinction remains central to forensic FGG practice: the database match guides the investigation, whereas final identification requires multidisciplinary evaluation and independent confirmation.

### 6.3. Broader Applications Related to SNP-Based Distant Kinship Inference

Although genetic genealogy originally emerged in non-forensic contexts such as ancestry exploration and unknown-parentage searches, FGG specifically refers to the forensic adaptation of these methodologies for law-enforcement and human-identification investigations [30,78]. Consequently, civil and administrative applications of genetic genealogy should be distinguished from operational FGG. Nevertheless, these adjacent applications remain relevant to the broader scope of this review because they illustrate how genome-wide SNP data, shared-segment analysis, and distant kinship inference can support biological relationship reconstruction when close relatives are unavailable.

Within this broader context, SNP-based distant kinship inference may contribute to unknown-parentage investigations, adoption-related searches, complex missing-person cases, historical identifications, and selected civil or administrative kinship questions. In contrast, inheritance disputes, citizenship claims, and military repatriation programs are more accurately characterized as kinship-comparison or human-identification contexts rather than FGG unless they specifically involve genetic genealogy database matching, genealogical reconstruction, and independent forensic confirmation [2,28,30,84]. This distinction is important because FGG primarily functions as an investigative lead-generation workflow, whereas formal kinship testing generally evaluates predefined relationship hypotheses using reference samples and statistical interpretation.

The analytical power of dense genome-wide SNP datasets enables increasingly distant biological relationships to be evaluated through identification of shared autosomal segments, although performance depends strongly on marker density, genotype quality, database composition, population structure, and the analytical framework employed (Figure 2, Table 2) [25,26,28,31,38,44]. Segment-based genetic genealogy searches generally require dense genome-wide datasets comprising hundreds of thousands of SNPs, whereas lower-density targeted forensic SNP panels may support defined kinship comparisons involving close or moderately distant relationships but should not be considered analytically equivalent to high-density genetic genealogy database matching [24,28,31,38,43,44,47,48]. These analytical distinctions are also relevant to immigration and refugee family-reunification proceedings, in which DNA-based relationship testing may be used to evaluate close biological relationships when documentary evidence is insufficient [85,86]. Although genome-wide SNP approaches could potentially support relationship assessment when only distant relatives are available, they should not be presented as an established routine replacement for validated STR-based testing. Any future or case-specific implementation should rely on validated decision thresholds, population-appropriate reference data, transparent reporting of uncertainty, and procedures that are understandable to applicants and legal representatives. Appropriate safeguards would also be required to minimize false inclusion or exclusion and to avoid conflating genetic kinship with legal, cultural, or social definitions of family.

Complementary marker systems such as microhaplotypes and compound markers may further strengthen forensic kinship analysis by increasing allelic diversity, improving performance with degraded DNA, and supporting interpretation in genetically diverse populations [17,87,88,89]. In addition, SNP-mixture deconvolution workflows are being developed to address mixed forensic samples, where multiple contributors may otherwise obscure kinship signals [90]. These approaches are best understood as complementary forensic-genomic developments rather than standard genetic genealogy database-matching methodologies.

Accordingly, SNP-based distant kinship inference extends beyond operational FGG. Its applications should be differentiated according to analytical objective, marker density, data quality, and interpretive framework. Genetic genealogy database matching, formal kinship testing, targeted SNP panels, low-coverage sequencing, SNP capture, microhaplotypes, and mixture-deconvolution workflows can all contribute to forensic relationship analysis, but they should not be treated as interchangeable methods or as providing equivalent evidentiary outputs. Maintaining these distinctions helps preserve the broader relevance of SNP-based kinship inference without overstating the scope of FGG itself.

**Table 2:** Comparison of SNP-based approaches for distant kinship inference and forensic relationship analysis.

Approach	Typical Analytical Context	Approximate Relationship Range	Key Observations
Conventional STR kits	Routine forensic identification and close-kinship testing	Primarily close relationships	STRs remain highly informative for individual identification and close kinship but have limited resolution for distant relationship inference because of the relatively small number of loci and higher mutation rates compared with SNPs [1,14,15].
100–200 independent SNPs	Parentage, sibship, and pedigree reconstruction under controlled conditions	Parent-offspring and full siblings; selected close relationships	Low-density independent SNP sets can support close relationship inference or pedigree reconstruction but are not designed for GG database matching [34,47,91,92].
~4000–10,000 targeted forensic SNPs	Targeted forensic kinship analysis and sparse SNP database searches	Up to third- or fourth-degree relationships under favorable conditions	Targeted panels such as ForenSeq™ Kintelligence (Verogen, Inc., San Diego, CA, USA) can support kinship estimation in defined forensic contexts, but their performance is not equivalent to dense genome-wide GG database matching [24,43,47,48].
Dense SNP arrays/Whole-Genome Sequencing	Segment-based genetic genealogy database matching and distant kinship inference	Useful for more distant relationship inference; performance varies by SNP density, DNA quality, database composition, endogamy, and genealogical record availability	High-density genome-wide profiles enable shared-segment detection, total shared cM estimation, and relationship-range inference in GG database searches. These outputs generate investigative leads rather than stand-alone forensic conclusions or formal identity determinations [23,25,28,31,38,44,93].
IBD coefficient- and segment-based statistical approaches	Formal distant relationship inference, simulation studies, and comparative evaluation of relationship-degree resolution	Variable; depends on relationship degree, marker density, recombination, and analytical model	IBD-based statistical approaches help evaluate the limits of relationship-degree classification and demonstrate why adjacent distant relationship classes may overlap. These approaches support interpretation of SNP-based distant kinship inference but should be distinguished from routine GG database matching [25,26,38,94,95].
SNP capture/low-coverage sequencing	Compromised remains, historical samples, low-input or low-coverage forensic material	Variable; depends on recovered SNPs, coverage, references, and analytical method	SNP capture and low-coverage sequencing methods can support identity or relatedness inference when microarray-quality profiles are unavailable, but they are best described as forensic SNP-based kinship approaches outside routine GG database matching [55,56].
Microhaplotypes, compound markers, and SNP-mixture workflows	Complementary forensic-genomic analysis, degraded DNA, genetically diverse populations, and mixture interpretation	Context-dependent	These approaches may increase informativeness or preserve kinship signal in difficult samples, but they should not be presented as standard FGG database-matching methods [17,87,88,89,90].

SNP: single-nucleotide polymorphism; STR: short tandem repeat; FGG: forensic genetic genealogy; GG: genetic genealogy; IBD: identity-by-descent; cM: centiMorgan. Relationship ranges are approximate and should not be interpreted as deterministic limits. Performance depends on marker density, DNA quality, population context, database composition, endogamy, recombination, genotyping error, and the analytical framework used.

## 7. Limitations and Challenges

A central limitation in the forensic implementation of SNP-based distant kinship inference is the difference between high-quality reference genotypes and SNP profiles generated from forensic evidence. Genetic genealogy databases are populated primarily with high-quality consumer or reference profiles, whereas forensic samples may be degraded, low-template, inhibited, contaminated, mixed, or otherwise incomplete. These conditions can reduce SNP recovery, increase missing data and genotyping error, fragment true IBD segments, and decrease the sensitivity of segment-based matching. Suitability for GG database searching should therefore be evaluated based on profile completeness, genotype error, platform compatibility, and the marker-density considerations summarized in Section 3 and Table 2 [24,28,38,44,55,56,61,62].

Beyond laboratory limitations, fundamental biological processes also constrain the resolution of distant kinship inference. As genealogical distance increases, the expected proportion of shared genetic material declines rapidly across generations. Consequently, the probability of detecting informative IBD segments between distant relatives decreases, and the genetic signal of relatedness may become difficult to distinguish from background genomic similarity within the population [26,43,96,97,98]. A related challenge arises from the stochastic nature of meiotic recombination and inheritance. The amount of genomic sharing between relatives may deviate substantially from theoretical expectations, producing overlapping distributions of shared cM values and IBD segment counts across adjacent relationship categories. Such overlap can complicate discrimination among distant relatives and introduce uncertainty when relationship hypotheses involve individuals separated by multiple generations [29,39,43,94,95].

Population structure, endogamy, founder effects, and pedigree collapse represent additional interpretive challenges, although their relevance differs according to the analytical framework employed. In formal kinship testing, population structure may be addressed through allele-frequency databases, population-specific parameters, kinship coefficients, or structured population models. In contrast, GG database matching generally does not correct match lists using equivalent formal population-genetic approaches; instead, genealogical interpretation is required to recognize patterns suggestive of endogamy, background relatedness, or population-specific overmatching [29,37,38,46,60,98]. Accordingly, population context should be considered primarily as an interpretive factor influencing genealogical reconstruction rather than as a parameter automatically incorporated into GG database matching.

The composition and representativeness of genealogical databases further influence the effectiveness of FGG investigations. Consumer genetic genealogy databases consist largely of voluntarily contributed profiles and remain unevenly distributed across global populations. GG databases are enriched for US-based participants with European ancestry, and representation is markedly limited for other countries and ethnic backgrounds [23,30,49,99]. This demographic imbalance may reduce match probabilities and genealogical resolution for underrepresented populations, potentially creating disparities in investigative success among different population groups.

Even when informative genetic matches are identified, successful interpretation requires careful integration of genetic, genealogical, and investigative information. Genetic matches rarely provide direct identification; instead, they function as probabilistic leads that must be contextualized through genealogical reconstruction and independent investigative evidence [29,49]. Incomplete historical records, pedigree inaccuracies, adoption, misattributed parentage, non-paternity events, migration, name changes, and incomplete public documentation may complicate genealogical reconstruction and introduce uncertainty into identity-hypothesis generation [29,49].

Ethical and legal considerations add a further layer of complexity to FGG implementation. Because genetic data are inherently relational, analysis of one individual’s DNA may indirectly reveal information about biological relatives who have not themselves consented to genetic testing [30,49,100]. This relational nature of genetic information raises important questions regarding privacy, informed consent, database governance, law-enforcement access, data retention, secondary data use, and the balance between investigative utility and individual or familial privacy [29,30,49,54,63,65,66,67,68,69,70,71,72,100]. Governance approaches differ substantially across jurisdictions and database platforms. In the United States, implementation has been strongly shaped by platform-specific consent models, law-enforcement access policies, and investigative guidelines. In the United Kingdom and the European Union, proportionality, privacy protection, data minimization, and oversight are more closely linked to human-rights and data-protection frameworks. In Australia and Canada, emerging casework experience and policy discussions have focused on missing-person and UHR investigations, database access, transparency, and public acceptability. Across these contexts, opt-in versus opt-out models, database platform policies, secondary use, data retention, cross-border access, oversight, and privacy implications for non-consenting relatives remain central governance issues [29,30,54,63,65,66,67,68,69,70,71,72].

Collectively, these limitations demonstrate that although SNP-based distant kinship inference has substantially expanded the capabilities of forensic genetics, its performance remains constrained by sample quality, marker density, recombination, stochastic inheritance, population structure, database composition, genealogical record availability, and governance frameworks. Maintaining clear distinctions among high-density GG database matching, lower-density forensic SNP panels, formal kinship testing, and complementary SNP-based approaches is essential to avoid overgeneralizing the performance or evidentiary significance of any single method.

## 8. Conclusions

Genome-wide SNP analysis has expanded forensic genetics from locus-based human identification toward distant kinship inference based on shared genomic segments. Dense SNP datasets can support IBD segment detection, shared-cM estimation, and relationship inference across degrees of relatedness that often exceed the practical resolution of conventional STR-based approaches. However, the value of SNP data depends strongly on marker density, genotype quality, population context, database composition, recombination, and the analytical framework used.

The central message of this review is that SNP-based distant kinship inference, genetic genealogy database matching, formal forensic kinship testing, and independent forensic confirmation are related but non-interchangeable processes. GG database matching primarily generates investigative leads through shared-segment metrics and genealogical reconstruction, whereas formal forensic kinship testing evaluates predefined hypotheses using reference samples and statistical interpretation. Targeted forensic SNP panels, SNP capture, low-coverage sequencing, Bayesian models, machine-learning classifiers, microhaplotypes, and mixture-deconvolution workflows may complement this broader field, but they differ in validation status, assumptions, outputs, and evidentiary meaning.

The effectiveness of SNP-based distant kinship inference remains influenced by recombination, stochastic inheritance, population structure, endogamy, database composition, marker density, genotype quality, and the availability of genealogical records. These challenges are amplified in forensic casework, where evidentiary samples may be degraded, low-template, mixed, or incomplete, while genealogy databases are populated primarily with high-quality consumer or reference genotypes. Uneven database representation across populations may further influence match probability and investigative success.

Future progress will require transparent validation, responsible use of genome-wide SNP data, improved methods for compromised forensic samples, and clearer standards for database matching, statistical interpretation, and independent confirmation. Maintaining conceptual boundaries among investigative leads, relationship hypotheses, and formal identification is essential for scientifically robust, ethically responsible, and legally defensible implementation of SNP-based forensic genomics.

## Data Availability

No new data were created or analyzed in this study. Data sharing is not applicable to this article.

## Abbreviations

The following abbreviations are used in this manuscript:

aiSNPs	Ancestry-informative SNPs
bp	Base pairs
cM	centiMorgans
CODIS	Combined DNA Index System
FGG	Forensic genetic genealogy
FIGG	Forensic investigative genetic genealogy
HID	Human identification
HMMs	Hidden Markov Models
IBD	Identical-by-Descent
IBS	Identical-by-State
IGG	Investigative Genetic Genealogy
iiSNPs	Identity-informative SNPs
liSNPs	Lineage-informative SNPs
LR	Likelihood Ratio
MPS	Massively Parallel Sequencing
mtDNA	mitochondrial DNA
PCR	Polymerase Chain Reaction
piSNPs	Phenotype-informative SNPs
SNP	Single Nucleotide Polymorphism
STR	Short Tandem Repeats
UHR	Unidentified Human Remains
WGS	Whole-Genome Sequencing
